# The Conserved ASCL1/MASH-1 Ortholog HLH-3 Specifies Sex-Specific Ventral Cord Motor Neuron Fate in *Caenorhabditis elegans*

**DOI:** 10.1534/g3.120.401458

**Published:** 2020-09-24

**Authors:** Lillian M. Perez, Aixa Alfonso

**Affiliations:** Biological Sciences, University of Illinois at Chicago, Illinois 60607

**Keywords:** Motor neurons, VCs, specification, differentiation, *hlh-3*

## Abstract

Neural specification is regulated by one or many transcription factors that control expression of effector genes that mediate function and determine neuronal type. Here we identify a novel role for one conserved proneural factor, the bHLH protein HLH-3, implicated in the specification of sex-specific ventral cord motor neurons in *C. elegans*. Proneural genes act in early stages of neurogenesis in early progenitors, but here, we demonstrate a later role for *hlh-3*. First, we document that differentiation of the ventral cord type C motor neuron class (VC) within their neuron class, is dynamic in time and space. Expression of VC class-specific and subclass-specific identity genes is distinct through development and is dependent on the VC position along the A-P axis and their proximity to the vulva. Our characterization of the expression of VC class and VC subclass-specific differentiation markers in the absence of *hlh-3* function reveals that VC fate specification, differentiation, and morphology requires *hlh-3* function. Finally, we conclude that *hlh-3* cell-autonomously specifies VC cell fate.

Cells in the nervous system, neurons and glia, are extremely diverse in shape, function, and the mechanisms by which they connect to other cells. Generation of neurons and their acquisition of unique features require the commitment to neural fate by an ectodermal descendant, the specification of neural class within the neuronal precursor, and the differentiation into unique transcriptomic and morphological states of the postmitotic cell. Importantly, the acquisition of unique neuronal class features, compared to pan-neuronal identity is seen to be regulated differently. Redundant regulators with multiple *cis*-regulatory inputs induce pan-neuronal features whereas terminal differentiation of neurons is induced by single inputs, encoded by so-called terminal selectors, and results in the expression of a unique repertoire of genes that promote neural class diversity (Hobert 2016b; Stefanakis *et al.* 2015). Thus, the neural diversity displayed by the nervous system is possible by the concerted action of terminal selector factors that function spatiotemporally with precision (Allan and Thor 2015; Hobert 2016a; Kratsios *et al.* 2017; Hobert and Kratsios 2019).

In *C. elegans*, the mechanisms that regulate neural specification can be studied thoroughly in time and in space, at single-cell resolution. This is a powerful model system that harbors a fully mapped body plan and nervous system, with continuously updated genomic and transcriptomic annotations, supporting studies in developmental biology, evolutionary conserved genes and networks, and beyond (Baker and Woollard 2019; Cooper and Van Raamsdonk 2018; Corsi *et al.* 2015; Emmons 2016; Hammarlund *et al.* 2018; Sulston and Horvitz 1977). The classes of motor neurons in the ventral cord of *C. elegans* are diverse and contain both sex-shared neurons and sex-specific neurons, with known positions, connectivity, and neurotransmitter fate. They provide an excellent target to address the mechanisms by which sex-shared and sex-specific neurons are specified, which are currently not well understood. Our work adds to this body of knowledge and implicates the product of the proneural gene *hlh-3*, HLH-3, in the development of the hermaphrodite sex-specific ventral cord motor neurons.

Here we characterize the role of a conserved proneural-like protein HLH-3, the ortholog of ASCL1, also known as the mammalian Achaete-Scute homolog-1 (MASH-1), in *C. elegans* nervous system development. HLH-3 contains a conserved basic helix-loop-helix (bHLH) domain, which is 59% (31/54) identical to MASH-1 and 61% identical to ASCL1 (33/54). HLH-3 heterodimerizes with the Class I bHLH transcription factor HLH-2, predicted ortholog of TCF3/TCF4/TCF12 (Kim *et al.* 2018; Krause *et al.* 1997). Our previous work has implicated HLH-3 in the terminal differentiation of the hermaphrodite-specific motor neurons, HSNs, a bilateral pair of neurons that function in the egg-laying circuitry (Schafer 2006; Doonan *et al.* 2008; Raut 2017). Work by others has shown that the gene *hlh-3* has diverse functions in the nervous system, it is necessary for the appropriate death of the sisters of the NSMs (Thellmann *et al.* 2003); it works in combination with other transcription factors to induce the serotonergic program in the HSNs, and moreover, its ortholog, ASCL1, can be a functional substitute (Lloret-Fernández *et al.* 2018); it promotes neurogenesis of I4 (Luo and Horvitz 2017); it co-regulates the initiation of expression of the terminal selector gene *ttx-3* (Murgan *et al.* 2015); and it regulates expression of the chemoreceptor gene *srh-234* (Gruner *et al.* 2016). The above studies implicated *hlh-3* in the development of different types of neurons; however, its role in sex-specific neuron development is not as well documented.

We were the first to report that *hlh-3* is expressed in the embryonically generated P cells, ectodermal-like precursors of all post-embryonically generated ventral cord motor neurons. We also showed that by the third larval stage (L3) expression of a truncated translational fusion *hlh-3* reporter was restricted to the ventral cord motor neuron type C (VC) class, a hermaphrodite sex-specific type of neuron (Doonan *et al.* 2008). This expression pattern is consistent with a role in neuroblast specification, a function of canonical proneural genes. However, it remained to be determined whether *hlh-3* had a function in the specification of the P cell descendants (including sex-shared as well as sex-specific). Here we report on the role of *hlh-3* in the differentiation of postembryonic ventral cord motor neurons. We show it is necessary for the acquisition and maturation of the hermaphrodite sex-specific VC class only.

The postembryonic ventral cord motor neurons are made up of both sex-specific and sex-shared neurons arising from the anterior descendants of ectodermal-like P blast cells (Pn.a) (Sulston and Horvitz 1977). After two additional cell divisions, the Pn.aap cells give rise to the sex-specific neurons of the ventral cord. In hermaphrodites, the P3-P8.aap cells give rise to VCs (Figure 1A and 1B), whereas in males Pn.aapa and Pn.aapp (where n = descendant of P3 to P11), give rise to the ventral cord neuron type CA and CP, respectively (Sulston *et al.* 1980). Their fate acquisition (generation) is influenced by positional cues (Hox genes), differential survival (programmed cell death), and sexual identity (VC *vs.* CA/CP). The VCs of the hermaphrodite are positioned in the midbody and make up six of the total eight sex-specific neurons. Equivalent lineage descendants (Pn.aap) of P1, P2, and P9-12 cells in hermaphrodites undergo programmed cell death (Clark *et al.* 1993). These neuron classes (VC, CA, and CP) provide an opportunity to study the molecular mechanisms that drive sex-specific neuron differentiation in the ventral nerve cord.

**Figure 1:**
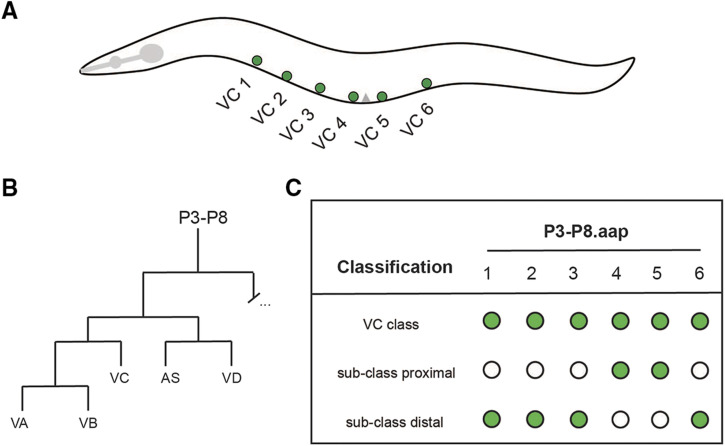
The ventral cord type C motor neuron class. A: Illustration of the position of the six VCs along the ventral nerve cord in the midbody region of an adult hermaphrodite. Anterior is to the left, ventral is down, and the gray triangle on the ventral surface indicates the location of the vulva. B: Diagram of the reiterative post-embryonic cell divisions produced by the P3.a to P8.a neuroblasts that give rise to the VCs (adapted from Sulston and Horvitz 1977) C: Diagram for VC classification includes two sub-classes: proximal (VC 4 and VC 5) and distal (VCs 1-3, 6). This classification format will be used throughout the rest of the figures.

Little is known about how VCs are generated and how they differentiate. To date, it is not known which factor(s) initiate the differentiation program of VCs to establish a class-wide identity. However, it is known that survival of VCs requires the function of the HOX gene *lin-39* and the HOX cofactors encoded by *unc-62* and *ceh-20* (Clark *et al.* 1993; Salser *et al.* 1993). UNC-62, along with LIN-39, promotes survival of the VCs by ensuring CEH-20 localizes to the nucleus; the LIN-39/CEH-20 complex then represses *egl-1* transcription (Liu *et al.* 2006; Potts *et al.* 2009). Sexual determination of the Pn.aap cells is established by the first larval (L1) stage (as VCs in hermaphrodites and the precursors of CAs and CPs in males) (Kalis *et al.* 2014). It was also shown that LIN-39 is not required for the expression of the VC terminal differentiation feature *ida-1*. Moreover, since the surviving descendants from P1, P2, and P9-12 still express *ida-1*::*gfp* in *lin-39**(lf)*; *ced-3**(lf)* double mutants, it was concluded that the role of LIN-39 is most likely restricted to VC survival, not differentiation (Kalis *et al.* 2014). However, recent evidence has implicated a role for LIN-39 in the expression of a VC marker *srb-16* (Feng *et al.* 2020).

While the mechanisms that regulate VC class specification have yet to be determined, the mechanisms that regulate VC subclass identity are better understood. Within the VC class, two VC subclasses are distinguished spatially by their proximity to the vulva, categorized as proximal VCs or distal VCs (Schafer 2006). The two VC neurons that flank the vulva are categorized as “proximal” (VC 4 and VC 5), whereas the other four VCs are “distal” to the vulva (VC 1-3, and VC 6) (Figure 1C). Genetic analysis of *unc-4* has revealed that the proximal VC subclass identity is determined by spatial cues. Specifically, the expression of *unc-4* as a VC proximal subclass identity gene requires the secretion of LIN-3/EGF (Epidermal Growth Factor) from vulval tissue (vulF cells) (Zheng *et al.* 2013). EGF signaling promotes proximal VC subclass fate by de-repression of *unc-4* in the proximal VCs only. Thus, a non-cell autonomous mechanism mediates one aspect of VC differentiation, specifically in proximal VCs.

Here we build on the current knowledge of neural specification in *C. elegans* and discover that the proneural-like bHLH factor, HLH-3, mediates specification and differentiation of the VC sex-specific motor neurons, that is, it is needed early and late in development. By using molecular markers to assess VC differentiation in the absence of *hlh-3* function, we find that VC class and subclass identity, as well as morphology, is compromised. Our work identifies a new role for the Achaete-Scute ortholog, HLH-3, in the ventral cord of *C. elegans*, that is the control of sex-specific motor neuron development. We conclude that HLH-3 is necessary for the expression of the earliest VC class-specific transcriptional regulator *(**lin-11**)* examined and is required for the expression of later acting VC class-specific genes.

## Materials And Methods

### Strain maintenance

All strains were maintained at 22° on nematode growth media using standard conditions (Brenner 1974). Some strains were provided by the CGC, which is funded by NIH Office of Research Infrastructure Programs (P40 OD010440). *hlh-3**(**tm1688**)* was isolated by the National Bioresource Project of Japan. *cccIs1* was kindly shared by Dr. Jennifer Ross Wolff, *uIs45* was kindly shared by Dr. Martin Chalfie, *otIs456* was kindly shared by Dr. Oliver Hobert, and both *otIs564* and *otIs619* were kindly shared by Dr. Paschalis Kratsios. See Supplemental Table 1 for a complete list of strains used in this study.

### Construction of transgenic strains

The transgenic strain harboring *icIs270* was generated by the integration of *akEx31** [pglr-5*::*gfp + **lin-15**(+)]* using UV-TMP treatment followed by outcrossing (see below). The VC rescue array *icEx274 [VC*::*hlh-3cDNA*::*GFP*; *pmyo-2*::*mCherry]* was generated by co-injection of the constructs pCFJ90 *(pmyo-2*::*mCherry)* and pRD2 *(VC*::*hlh-3cDNA*::*GFP)* into the mutant strain harboring *hlh-3**(**tm1688**)*; *otIs456** [pmyo-2*::*GFP*; *plin-11*::*mCherry]* at 20 ng/microliter and 2 ng/microliter, respectively. pRD2 was generated by Dr. Ryan Doonan to address whether *hlh-3* could rescue the egg-laying defective phenotype in *hlh-3**(**tm1688**)* (Doonan 2014). The pRD2 construct contains a VC specific promoter obtained from the vector pDM4, kindly provided by Dr. Michael Koelle driving expression of a *hlh-3* cDNA (Doonan 2014).

### Integration of extrachromosomal arrays

The transgenic strain harboring *icIs270* was generated by exposing L4 hermaphrodites to UV-TMP (350microJoules × 100 on Stratagene UV Stratalinker; 0.03 microgram/microliter TMP. Irradiated animals were placed onto seeded NGM plates and transferred the next day to fresh seeded NGM plates (3 Po/plate). These were followed to clone F1s (∼150) and subsequently to clone three F2s per F1.

### Construction of HLH-3::GFP CRISPR-Cas9 engineered line hlh-3(ic271)

Construction of the CRISPR-Cas9 engineered line required modification of two plasmids: the single guide RNA or sgRNA plasmid, pDD162 (Addgene #47549), and the repair template plasmid, pDD282 (Addgene #66823) (Dickinson *et al.* 2015). The target sequence GCTATGATGATCACCAGAAG was selected using the CRISPR design tool on Flybase consisting of a high optimal quality score (96). The sgRNA was cloned into pDD162 to create pLP1. The 5′ homology arm was designed as a gBlock containing a silent mutation at the PAM site to prevent Cas9 off-targeting. The gBlock was PCR amplified with primers acgttgtaaaacgacggccagtcgccggca and CATCGATGCTCCTGAGGCTCCCGATGCTCC and cloned into pDD282. The 3′ homology arm was designed via PCR using the primers CGTGATTACAAGGATGACGATGACAAGAGATAATCTGTTAAGTTGTACC and ggaaacagctatgaccatgttatcgatttccaaggagctggtgcacaag. The PCR product was purified and cloned into pDD282 to create pLP2. The modified constructs pLP1and pLP2, as well as the co-injection plasmid pGH8 (Addgene #19359) were co-injected into an N2 strain: sg-RNA plasmid (pLP1) at 50ng/uL; *hlh-3* repair template plasmid (pLP2) at 10ng/uL, and pGH8 at 2.5ng/uL. Screening was carried out according to the published protocol (Dickinson *et al.* 2015).

### Microscopy

Animals were mounted on 3% agarose pads containing droplets of 10mM levamisole. Fluorescent images were acquired with AxioVision on Zeiss Axioskop 2 microscope. Following the collection of images, some conversions were made with FIJI version 2.0.0 (grayscale images were converted with Lookup tables, Red or Green, and merged using Merge Channels) and processed into Adobe Illustrator version 23.1.1 for formatting. Fluorescent reporters were observed under confocal microscopy for the detection of a fluorescent protein signal (presence or absence) in transgenic lines. This study does not report quantification of intensity for any fluorescent reporter observed.

### Data availability

Strains and plasmids are available upon request. The authors affirm that all data necessary for confirming the conclusions of the article are present within the article, figures, and tables. Supplemental material available at figshare: https://doi.org/10.25387/g3.12996965.

## Results

### The Class II bHLH protein HLH-3 is expressed and localized to the nuclei of VCs from L1 through adulthood

**Figure 2:**
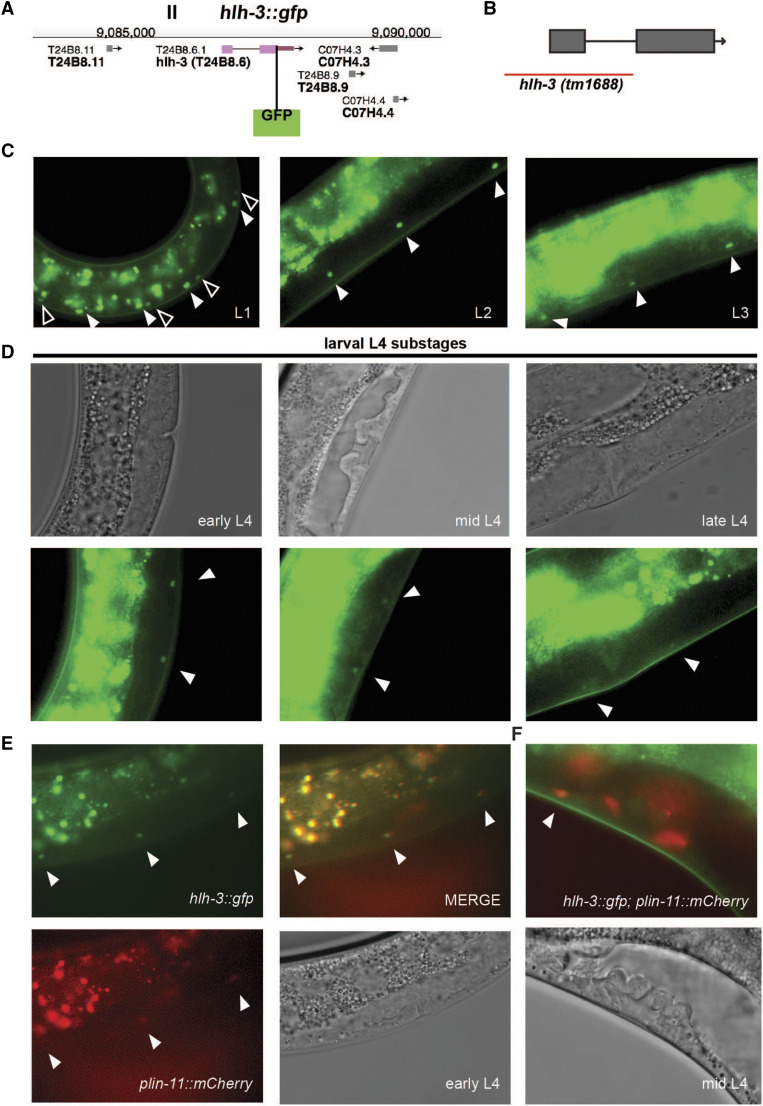
HLH-3 is first detected in nuclei of the Pn descendants (Pn.a and Pn.p) and becomes restricted to the nuclei of VCs as development proceeds. A: Diagram of the CRISPR-Cas9 engineered C-terminal GFP insertion at the *hlh-3* locus *hlh-3**(**ic271** [hlh-3*::*gfp])*. B: The *hlh-3**(**tm1688**)* allele represents a 1242 bp deletion that spans chromosome II from 35,589 to 36,831 and removes exon 1. Removal of this region, including most of the bHLH domain, results in a null allele (Doonan *et al.* 2008). C: Representative images of the midbody ventral cord of hermaphrodites harboring *hlh-3*::*gfp* at different larval developmental stages (L1, L2, and L3). At L1, filled arrowheads point to larger, more intense nuclei, presumably the Pn.a blast cells whereas the outlined arrowheads point to the diminishing expression in Pn.p blast cells (left panel). Filled arrowheads in L2 and L3 represent expression in VC nuclei (middle and right panels). Intense gut autofluorescence is apparent dorsally, above the ventral cord, because of overexposure in order to capture low levels of HLH-3::GFP. D: Representative images of the midbody ventral cord of hermaphrodites harboring *hlh-3*::*gfp* over distinct L4 developmental stages (early, mid, and late). Larval substages (top panels) are classified by vulva morphology (Mok *et al.* 2015). Intense gut autofluorescence is apparent above the ventral cord because of overexposure in order to capture low levels of HLH-3::GFP. Filled arrowheads point to the proximal VCs (bottom panels). E: Overlapping expression (merge, top right) of *otIs456*, containing the VC marker *plin-11*::*mCherry* (bottom left), and *hlh-3*::*gfp* (top left), in an animal at the L4 molt (bottom right). Filled arrowheads point to co-labeled proximal VCs. F: Overlapping expression (merge) of *otIs456*, and *hlh-3*::*gfp* in a mid L4 animal. Filled arrowhead points to co-labeled proximal VCs. Expression of *otIs456* is also detectable in vulval cells.

We have previously shown that in hermaphrodites, *hlh-3* is expressed in the postembryonic descendants of the ectodermal-like P cells as well as the HSNs (Doonan *et al.* 2008). We also have shown that *hlh-3* function is cell-autonomously required for normal axon pathfinding and terminal differentiation of the HSNs (Doonan 2014; Doonan *et al.* 2008; Raut 2017). In those studies, analysis of the expression of a translational fusion reporter with only the first eight amino acids of HLH-3 fused to GFP revealed that expression was widespread in the Pn.a descendants, dynamic, and with time, restricted to the VCs (Pn.aap) and HSNs. To confirm the endogenous spatiotemporal expression pattern of *hlh-3* we created *hlh-3**(**ic271**[hlh-3*::*gfp])* [herein called *hlh-3*::*gfp]*, a CRISPR-Cas9 engineered fluorescent tag at the C terminus of the *hlh-3* genomic locus (Figure 2A) following established genome-engineering protocols (Dickinson *et al.* 2015). The gene *hlh-3* is composed by two exons, the C terminal tag was engineered after the last amino acid in exon 2. The allele *hlh-3**(**tm1688**)* is a null allele that eliminates the majority of the bHLH domain and transcription start site (Figure 2B). Our analysis dissects both *hlh-3*::*gfp* endogenous expression and the consequence of the absence of *hlh-3* function in the context of the ventral cord motor neurons and primarily the VCs. First, in characterizing *hlh-3*::*gfp*, our analysis supports both our initial findings (Doonan 2014; Doonan *et al.* 2008), the recently reported observation that *hlh-3* expression reappears in the HSNs at the L4 developmental stage (Lloret-Fernández *et al.* 2018), and expands our understanding of its role in the VCs (Doonan 2014; Raut 2017).

We confirmed that *hlh-3* is expressed post-embryonically in the P cells and their descendants and becomes restricted to the terminally differentiated VCs present in adults. After hatching, animals show the expression of *hlh-3*::*gfp* in the descendants of the P cells throughout the ventral nerve cord (VNC). We highlight the expression of *hlh-3*::*gfp* in an early L1 animal wherein Pn.p expression extinguishes faster than that in Pn.a and its descendants (Figure 2C, left panel). As development proceeds (L2 and L3), expression is extinguished from other descendants of the Pn.a cells and restricted to the VCs (Figure 2C, middle and right panels). While fluorescent reporter intensity was not quantified, *hlh-3*::*gfp* expression appears to be down-regulated in a window of the fourth larval stage (L4) development ranging from mid L4 to late L4, before increasing in adulthood (Figure 2D, middle and right panels). To ensure that the detected nuclei are those of VCs, we characterized whether there was co-expression of *hlh-3*::*gfp* with a known VC marker, *plin-11*::*mCherry (**otIs456** [pmyo-2*::*GFP*; *plin-11*::*mCherry])* (Figure 2E, bottom left). We find that the *hlh-3*::*gfp* positive nuclei are also *plin-11*::*mCherry* positive throughout the L4 developmental stage (Figure 2E and 2F). Interestingly, low levels of *hlh-3*::*gfp* expression is also observed in a pair of vulval cells during mid-late substages of L4 development, suggesting a role for *hlh-3* in these lineages (data not shown). Expression of *hlh-3*::*gfp* in VCs from their birth in L1 through their terminally differentiated stage in adulthood prompted us to investigate the role of *hlh-3*, as a factor required for an early role in promoting VC fate and required for maintenance of VC fate throughout development. Throughout we will use the allele *hlh-3**(**tm1688**)*, further referred to in this paper as *hlh-3**(lf)*.

### The differentiation of VC class and VC subclass motor neurons is dynamic

Before we analyzed the role of *hlh-3* in VCs, we first characterized differentiation features of VCs by defining VC class *vs.* VC subclass-specific terminal identity. Here we took advantage of fluorescent transcriptional reporter genes that serve as markers of VC fate. We examined the expression of the known VC class-specific markers *lin-11*, *ida-1*, and *glr-5* encoding a LIM homeodomain transcription factor (Freyd *et al.*1990), a protein tyrosine phosphatase-like receptor protein homolog of IA2 (Cai *et al.* 2001, 2004; Zahn *et al.* 2001), and a glutamate receptor subunit (Brockie *et al.* 2001), respectively. We confirmed that expression of *lin-11* in VCs is observed as early as the second larval stage (L2), and through adulthood (data not shown) (Hobert *et al.* 1998; Zheng *et al.* 2013).

**Figure 3:**
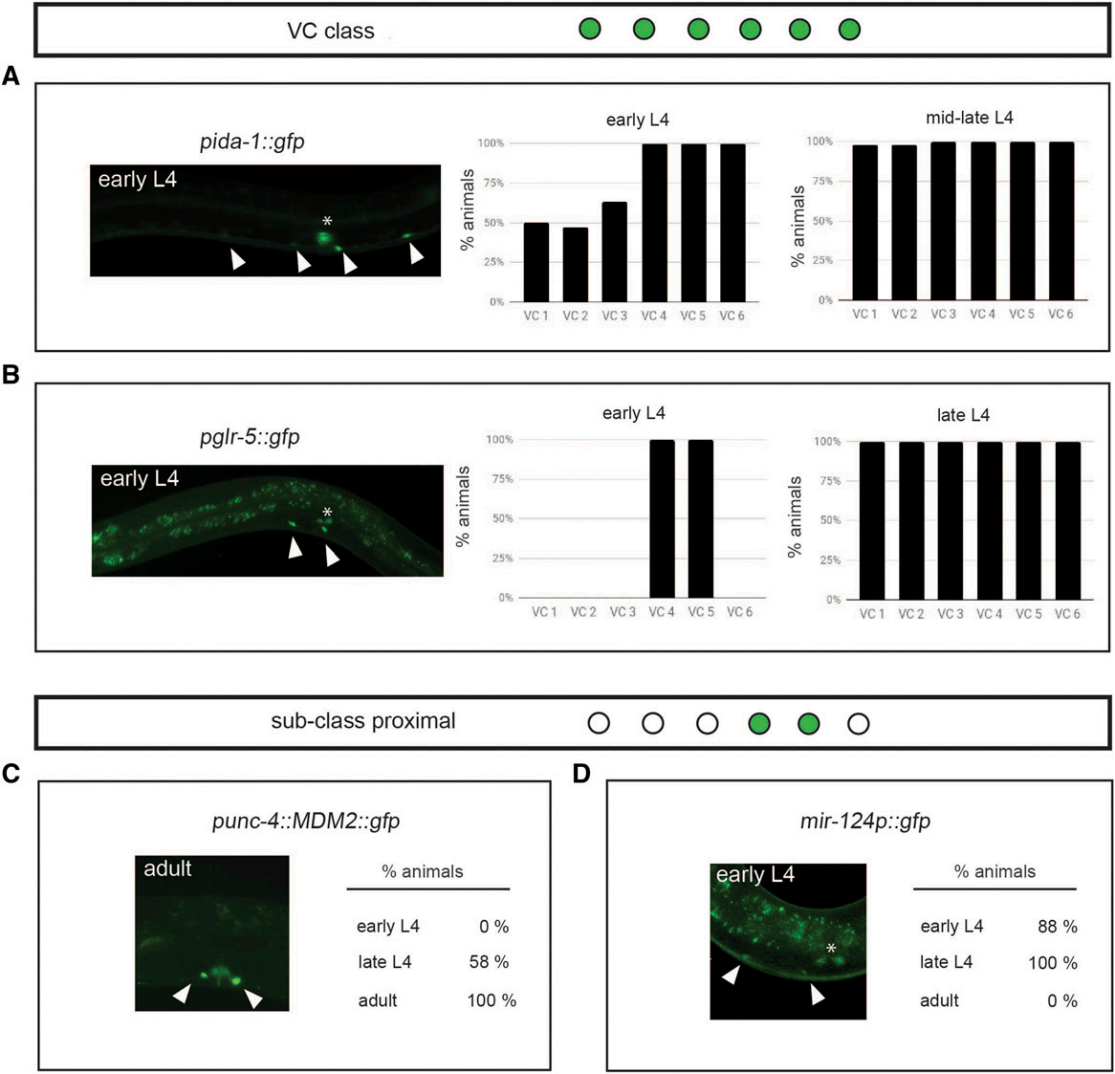
The spatiotemporal expression of VC class and subclass-specific identity features is dynamic. A: Expression pattern of the VC class differentiation feature *ida-1* in early and mid-late L4 developmental stages. Representative early L4 hermaphrodite expresses *inIs179** [pida-1*::*gfp]* in four VCs, VC 3 – VC 6 (indicated by arrowheads). Graphs report the percent of animals expressing *inIs179** [pida-1*::*gfp]* (early L4, n = 20; mid-late L4, n = 40) in each VC. Since all VCs express *pida-1*::*gfp* by mid-L4, quantification of *pida-1*::*gfp* expression for mid and late L4 stages is pooled. Asterisk highlights expression in the uV1 cells too. B: Expression pattern of the VC class differentiation feature *glr-5* in early and late L4 development. Image shows an early L4 hermaphrodite expressing *icIs270** [pglr-5*::*gfp]* in the proximal VCs (indicated by arrowheads). Graphs report the percent of animals expressing *icIs270* in early L4 (n = 10), mid L4 (n = 15), and late L4 (n = 15) developmental stages in each VC. Asterisk highlights expression in the HSNs too. C: Quantification of expression of the VC subclass feature, *unc-4*, from L4 development through adulthood. Image shows the expression of *uIs45** [punc-4*::*MDM2*::*GFP]* in an adult (indicated by arrowheads). The percent of animals expressing the VC subclass marker in both cells (VC 4 and VC 5) at early L4 (n = 8), late L4 (n = 12), and adult (n = 19) stages is listed adjacent to the image. D: Quantification of expression of VC subclass feature, *mir-124*, from L4 development through adulthood. Image shows expression of *mjIs27** [mir-124p*::*gfp + **lin-15**(+)]* in the proximal VCs of an early L4 hermaphrodite. The percent of animals expressing the VC subclass marker in both cells (VC 4 and VC 5) at early L4 (n = 8), late L4 (n = 13), and adult (n = 10) substages is listed adjacent to the image. Asterisk highlights expression in the HSNs too.

Unlike *lin-11*, a transcriptional regulator, the expression of the VC class terminal identity genes *ida-1* and *glr-5*, was only detected later in development, arising at the L4 developmental stage (Figure 3A and 3B). Analysis of these VC class differentiation markers throughout L4 substages revealed distinct spatiotemporal patterns suggesting different pathways regulate them. Expression of *ida-1* and *glr-5* is not equivalent across all six VCs during L4 development (Figure 3A and 3B). Classification of the L4 substages (early, mid, and late) is based on the vulval L4 morphology as previously described (Mok *et al.* 2015). We noted that *ida-1* expression in early L4 development is robust in the posterior VCs (VC 4-6), whereas, the anterior set of VCs show expression in only half of the sampled individuals. By mid L4, all individuals express *ida-1* equivalently in VCs (100% animals); therefore, the individuals in mid and late substages are grouped together (Figure 3A, right panel). In contrast to *ida-1*, *glr-5* expression is restricted to the proximal VC subclass in the early L4 substage and only fully expressed in all six VCs in the late L4 substage (Figure 3B). Thus, while the six VCs terminally express their class-specific terminal differentiation genes *ida-1* and *glr-5*, the initiation of transcription is distinct across the sub-stages of L4 development.

Next, we characterized the expression pattern of the VC subclass-specific terminal identity genes *unc-4* and *unc-17*. Others have shown that *unc-4* expression requires *lin-11* and vulval EGF signaling (Zheng *et al.* 2013). We corroborate that *unc-4* expression is detected after the mid-L4 stages and is maintained throughout adulthood only in VC 4 and VC 5 (Figure 3C). The expression of UNC-17, in turn, is known to require a posttranscriptional step mediated by UNC-4 (Lickteig *et al.* 2001). Therefore, we analyzed the expression of two transcriptional *unc-17* reporters. To our surprise, and in contrast to work by others, we only detect the expression of these *unc-17* reporters in VC 4 and VC 5 at the adult stage regardless of which reporter we characterized (Supplemental Figure 1A-E, and F top left panel). However, our work is different from others (Pereira *et al.* 2015) in that we did not assess a translational reporter. Instead, we looked at two transcriptional reporters *vsIs48** (punc-17*::*gfp)* and *mdEx865 (unc-17p*::*NLS*::*mCherry + **pha-1**(+))* and did not observe *unc-17* expression in the distal VCs 1-3 and 6 with either reporter. Although we do not see the *unc-17* reporters in the distal VCs, we do see expression in proximal VCs, as expected. However, expression is not always detectable in both proximal VCs in exactly the same individual (Supplemental Figure 1D and 1F). Our observations are also consistent with previous reports that anti-UNC-17 immunoreactivity is robust in VC 4 and VC 5, but rarely detectable in distal VCs (Lickteig *et al.* 2001; Duerr *et al.* 2008) and possibly only in the second larval (L2) stage (Alfonso *et al.* 1993).

### mir-124 is a novel VC subclass-specific identity feature

**Figure 4:**
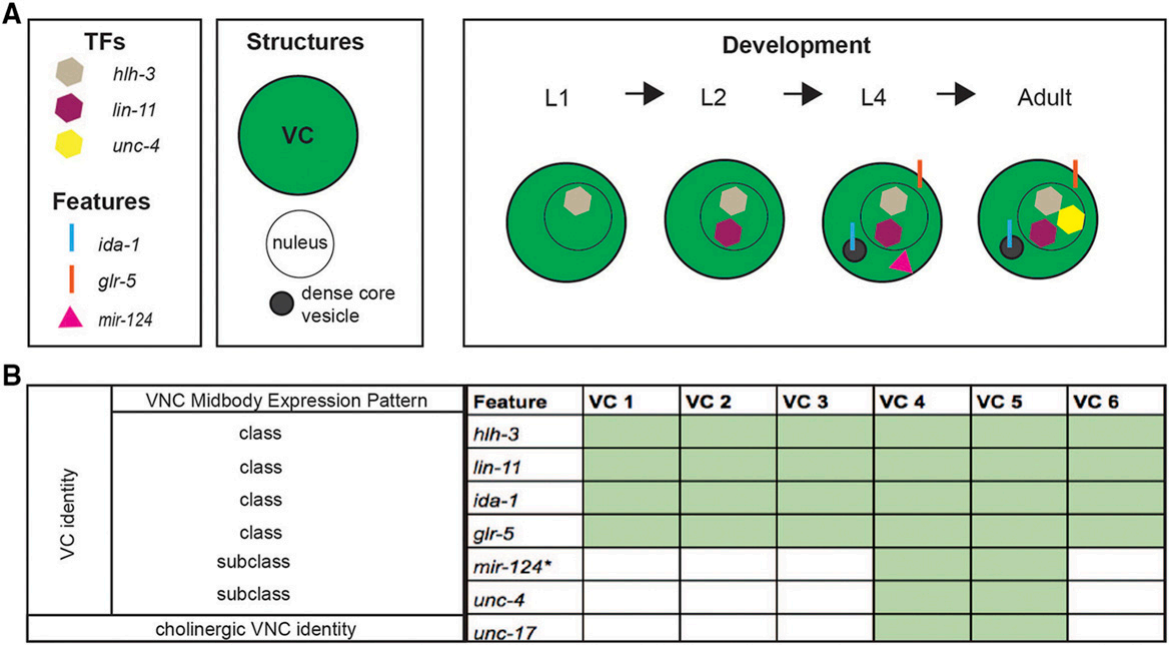
Summary of VC class and subclass identity. A: Diagram of genes encoding transcription factors (TFs) and class or subclass-specific features and structures expressed in VCs throughout post-embryonic development. B: Summary of the expression pattern of distinct VC class and VC subclass identity features in the midbody of the ventral cord of the hermaphrodite. While *unc-17* is expressed in the subclass proximal VCs, it is also expressed in all VNC cholinergic motor neurons, therefore not VC specific. Our analysis is primarily based on the expression of integrated transcriptional reporters (See Supplemental Table 1 for the list of strains containing these markers). With the exception of *mir-124*, all reported features are maintained through adulthood (denoted by an asterisk).

To expand the molecular repertoire of VC class and subclass markers we looked at several other genes known to be expressed in VCs (*flp-11*, *ntc-1**)*. However, *flp-11* and *ntc-1* expression is not unique to VCs in the ventral cord (Kim and Li 2004; Garrison *et al.* 2012). Furthermore, we examined the list of predicted targets of HLH-3/HLH-2 heterodimers (Grove *et al.* 2009) to identify genes that may be expressed in VCs too. The gene *mir-124* appeared as a logical candidate because it is a predicted target of *hlh-3* (Grove *et al.* 2009), and its expression is detected in a variety of sensory neurons, as well as the CEMs and HSNs, sex-specific neurons in males and hermaphrodites, respectively (Clark *et al.* 2010). We found *mir-124*, the highly conserved non-coding microRNA, as a novel VC subclass-specific differentiation feature. We characterized *mjIs27* [*mir-124p*::*gfp + **lin-15**(+)]* expression across postembryonic development, and only see it in a restricted window. We found *mir-124* is expressed from early L4 larval substages through early adulthood, but not in mature gravid egg-laying hermaphrodites (Figure 3D), which suggests it may be required for the maturation of the VCs but not for maintenance of VC fate. This expression pattern is unlike that of other proximal VC identity features, *unc-4* and *unc-17*, which are expressed throughout adulthood (Figure 3C; Supplemental Figure 1). Therefore, we classify *mir-124* as a novel VC subclass-specific feature expressed during early differentiation. In summary, we conclude that *mir-124* can be added to the list of VC identity features belonging to the proximal class (Figure 4).

### Classification of VC identity

Thus far, we have shown that the VC class of neurons acquire class-specific features via mechanisms that differ in time and space. We have also shown that not all six VCs are identical in their repertoire of transcriptional activity. In Figure 4, we summarize the spatiotemporal expression pattern of VC identity features. Two genes encoding presumptive transcription factors, *hlh-3* and *lin-11*, are VC class-specific and *hlh-3* expression precedes that of *lin-11* (Figure 4A-C). We classify the *ida-1* and *glr-5* genes as VC class-specific as well, as they are observed in all six VCs from L4 through adulthood. In contrast, *mir-124* is not expressed in adulthood, as the rest of the VC terminal identity features are. Finally, *unc-17* was observed in the proximal VCs only. It is worth emphasizing that aside from the analysis of the CRISPR-Cas9 engineered *hlh-3*::*gfp* line, our analysis is predominantly based on the characterization of transcriptional reporters (Figure 4; Supplemental Table 1).

### hlh-3 function is required for the acquisition of VC class and VC subclass identity features

**Figure 5:**
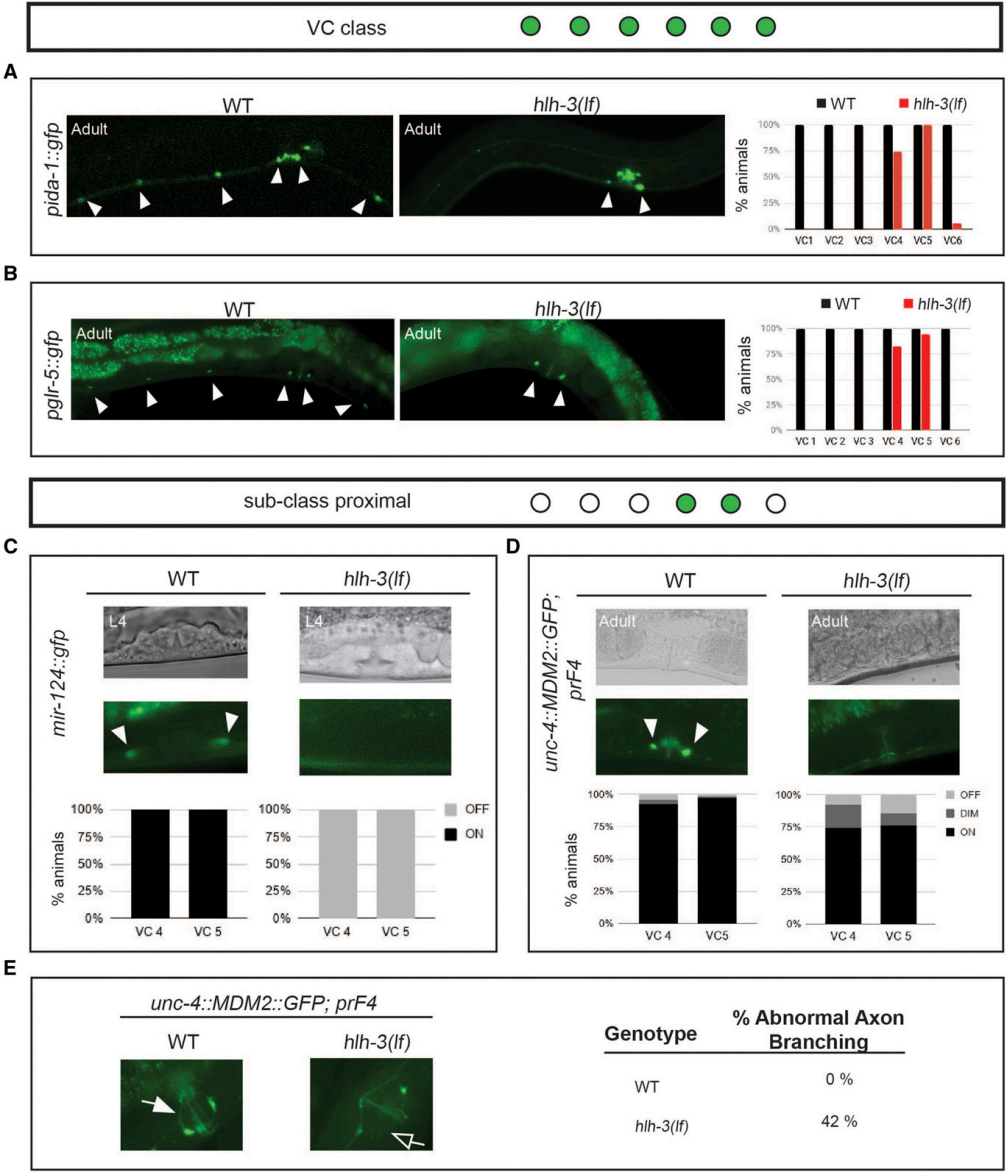
VCs require HLH-3 to acquire class-specific and subclass-specific differentiation features and normal axon morphology. A-B: Representative images of WT or *hlh-3**(lf)* one-day-old adults harboring the indicated reporters. Filled arrowheads point to detectable VCs in either genotype. Graphs report the percent of animals expressing each reporter in each VC of WT (black bars) and *hlh-3**(lf)* (red bars) one-day-old adults. The expression of the *ida-1* marker (*inIs179*) was quantified in WT (n = 15) and *hlh-3**(lf)* (n = 35) individuals (panel A). The expression of the *glr-5* marker (*icIs270*) was quantified in WT (n = 15) and *hlh-3**(lf)* (n = 30) individuals (panel B). C-D: Representative images of WT and mutant hermaphrodites at different stages of development and harboring the indicated reporters of VC subclass features *mir-124** (**mjIs27**)* and *unc-4** (**uIs45**)*. Fluorescent images of the vulval region of DIC imaged hermaphrodites (top panels) only revealed expression in the proximal VCs of WT individuals (indicated by filled arrowheads). Quantification of the percent of animals with detectable reporter expression of *mir-124* (*mjIs27*) in VC 4 or VC 5 is reported in the graph below the images. Fluorescence in mid-L4 hermaphrodites of the indicated genotypes and harboring *mjIs27* was either detectable (on) or not detectable (off); WT mid L4s (n = 17) and *hlh-3**(lf)* mid L4s (n = 14). Quantification of the percent of animals with detectable reporter expression of *uIs45* reporter expression in VC 4 or VC 5 is shown in the graph below the images. Fluorescence was either bright (on), dim, or not detectable (off) for expression of *uIs45* in WT (n = 66) and *hlh-3**(lf)* (n = 61) one-day-old adults. E: Quantification of proximal VC axon branching in WT and *hlh-3**(lf)* individuals. Normal axons branch into a vulval ring, as observed with *uIs45* in the WT genotype highlighted by the filled arrows (left panel). In contrast, *hlh-3**(lf)* hermaphrodites display abnormal axon branching highlighted by the unfilled arrows (right panel). The numbers to the right represent the percent of individuals with abnormal branching in one-day-old adult WT (n = 15) and *hlh-3**(lf)* (n = 24) hermaphrodites.

Previously, *hlh-3* has been shown to be required for HSN terminal differentiation (Doonan *et al.* 2008; Raut 2017; Lloret-Fernández *et al.* 2018). To address whether *hlh-3* has a role in VC differentiation we first examined reporters of VC terminal identity the genes *ida-1* and *glr-5*, in *hlh-3**(lf)* (Figure 5). We find that expression of the terminal VC class markers *ida-1* and *glr-5* is eliminated in the distal VCs of one-day-old *hlh-3**(lf)* adult hermaphrodites, synchronized as L4s the day before (Figure 5A and 5B). A defect in the expression of these differentiation markers is less severe in the proximal VCs, VC 4 differentiation is slightly affected but VC 5 differentiation is almost like WT. However, since we show that *ida-1* and *glr-5* expression is detectable in early L4 wild type individuals (Figure 3A and 3B) we also examined their expression in *hlh-3**(lf)* L4 hermaphrodites. We find that expression of the differentiation features *ida-1* (Supplemental Figure 2) and *glr-5* (not shown) in the earlier stages of L4 development (L4.0 to L4.3) is completely absent in *hlh-3**(lf)* hermaphrodites. This indicates that from mid L4 through adulthood, an *hlh-3* independent mechanism regulates expression of these genes in the proximal subclass.

Next, we examined the expression of VC subclass-specific identity features, *mir-124*, *unc-4*, and *unc-17* (Figure 5; Supplemental Figure 1). We find that the early differentiation subclass-specific feature *mir-124** (**mjIs27** [mir-124p*::*gfp + **lin-15**(+)])* is completely absent in *hlh-3**(lf)* (Figure 5D). We followed up with an analysis of *unc-4* expression. Others have shown that the expression of this VC subclass-specific terminal identity gene is de-repressed in WT animals after EGF signaling in mid-L4 development (Zheng *et al.* 2013). Here, we find that the absence of *hlh-3* function reduces the frequency of *unc-4* expression (Figure 5E). Since *unc-4* expression is required for *unc-17* expression (Lickteig *et al.* 2001), not surprisingly we find that expression of *unc-17* is missing the proximal VCs in *hlh-3**(lf)* individuals (Supplemental Figure 1F).

### hlh-3 is required for normal axon branching of proximal VCs

Consistent with the reduced expression of VC terminal identity molecular markers *in **hlh-3**(lf)*, proximal VCs have abnormal axonal branching in the vulval ring (Figure 5F). This defect suggests that proximal VC function may be impaired in *hlh-3**(lf)*, as axonal branching is required for synaptic connections to the egg-laying circuitry (Schafer 2006). Thus, growth and maturation of VC axons require *hlh-3* function, as it is the case for the HSNs (Doonan 2014; Doonan *et al.* 2008; Raut 2017).

### VCs survive in the absence of hlh-3 function

**Figure 6:**
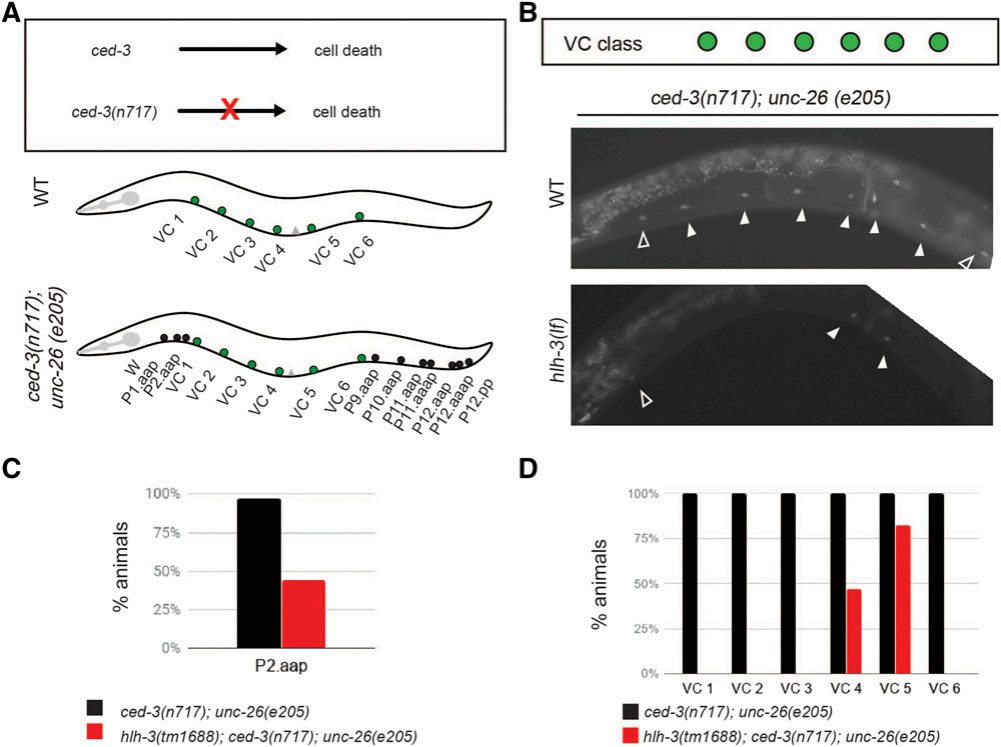
VCs do not inappropriately undergo programmed cell death (PCD) in the absence of *hlh-3* function. A: Diagram of outcome in the presence and absence of *ced-3* function. The presence of *ced-3* function in WT individuals results in PCD, the absence of *ced-3* function in the null allele *ced-3*(*n717*) prevents PCD. In the ventral nerve cord of WT animals, only the descendants of P3.aap to P8.aap or VCs report the expression of VC markers. However, in *ced-3**(**n717**)* nulls, the VC-equivalent descendants of P1 and P9-12, that normally undergo PCD, do not undergo PCD and report expression of VC markers. B: Representative images of *ced-3**(**n717**)*; *unc-26**(**e205**)* individuals with (WT), or without [*hlh-3**(lf)*] function. The *glr-5* VC marker (*icIs270*) was utilized to monitor the presence of VCs (filled arrowheads) and VC-like surviving cells (outlined arrowheads; specifically, P2.aap and P9.aap). The reporter *icIs270* is only detected in the proximal VCs (filled arrowheads) and a VC-like cell (outlined arrowhead; P2.aap) in the double mutant *hlh-3**(lf)*; *ced-3**(lf)*. Additionally, the reporter marks the HSNs. C: Quantification of the percent of one-day-old adults expressing *icIs270* in P2.aap in *ced-3**(**n717**)*; *unc-26**(**e205**)*; *pglr-5*::*gfp* (n = 35), and *hlh-3**(lf)*; *ced-3**(**n717**)*; *unc-26**(**e205**)*; *pglr-5*::*gfp* (n = 34). D: Quantification of the percent of one-day-old adults expressing *icIs270* in each VC of *ced-3**(**n717**)*; *unc-26**(205)*; *pglr-5*::*gfp* (n = 35) and *hlh-3**(lf)*; *ced-3**(**n717**)*; *unc-26**(**e205**)*; *pglr-5*::*gfp* (n = 34).

Our analysis of VC class and VC subclass markers indicate that the expression of VC differentiation markers is compromised in *hlh-3**(lf)* individuals. To ensure VC survival occurs we next sought to eliminate the possibility that VCs degenerate via necrosis or inappropriately undergo programmed cell death in *hlh-3**(lf)*. In *C. elegans* necrotic-like cell death results from excess Na^+^ influx through abnormal sodium permeant channels or gain of function mutations in Gα_s_ (Xu *et al.* 2001). Programmed cell death (PCD) is a conserved pathway executed by CED-3, a caspase that functions as the final determinant in the cell death pathway (Conradt *et al.* 2016). Inhibition of this pathway, by impairment of *ced-3* function, results in the survival of cells destined to die. In the context of the ventral nerve cord, the cells P1-P2.aap and P9-12.aap will survive (Figure 6A). Therefore, we introduced a *ced-3**(**n717**)* null mutation into *hlh-3**(lf)* mutants and analyzed the expression of a VC differentiation marker, *glr-5*, in *ced-3**(lf)* and *ced-3**(lf)*; *hlh-3**(lf)* individuals. Unlike *ced-3**(lf)* hermaphrodites, which express *glr-5* in all VCs, including the surviving P2.aap cell, we find that *ced-3**(lf)*; *hlh-3**(lf)* mutants do not express *glr-5* in most VCs. This defect is most notable in the distal class, but it is also apparent in the surviving VC-like cell P2.aap, which appears to follow the terminal differentiation program of the proximal VCs (Figure 6B-D). Therefore, we conclude that the reason VC neurons do not express *glr-5* in the absence of *hlh-3* function is that they need HLH-3 to fully differentiate and not because they undergo inappropriate PCD.

### hlh-3 functions cell-autonomously in the VC class

**Figure 7:**
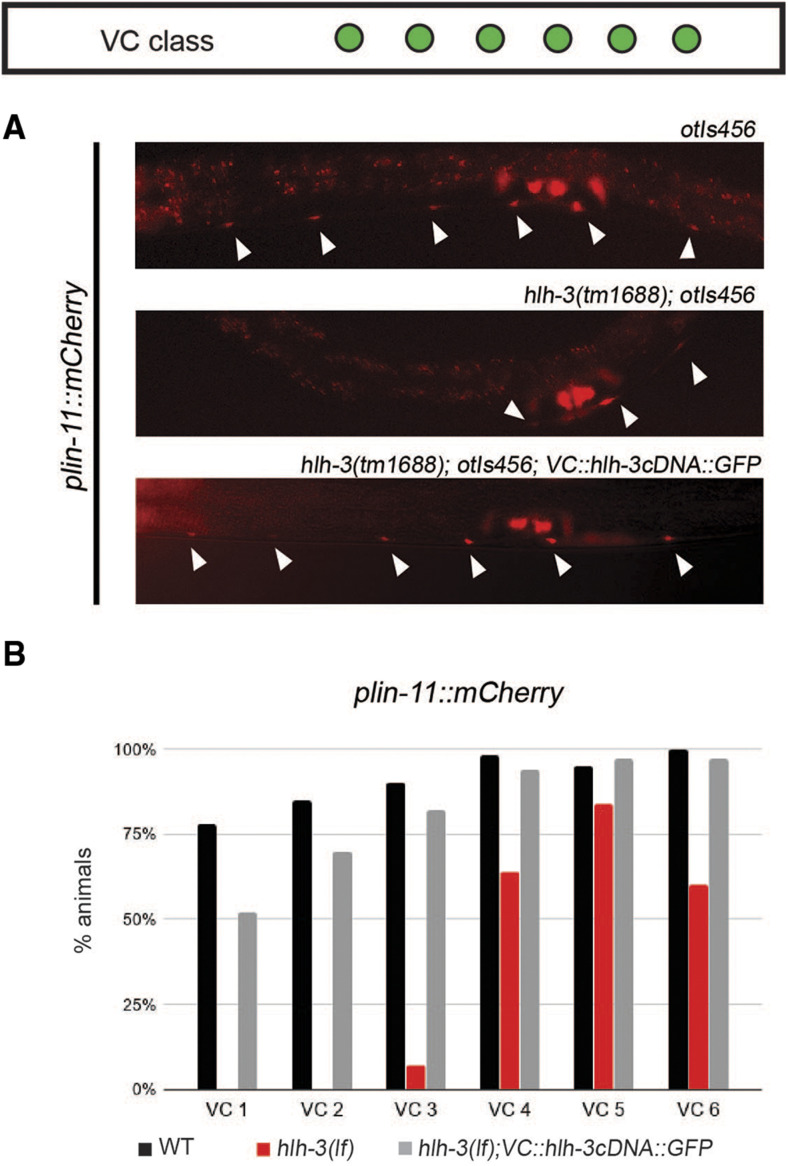
The function of *hlh-3* in VCs is cell-autonomous. A: Representative images of individuals harboring the *lin-11* marker (*plin-11*::*mCherry*) in WT (top panel), *hlh-3**(lf)* (middle panel), and VC-specific rescued lines (bottom panel). The reporter is normally expressed in all VCs (top panel, filled arrowheads). B: Quantification of the percent of mid-late L4 animals expressing *plin-11*::*mCherry* in each VC of WT (n = 41), *hlh-3**(lf)* (n = 48), and *hlh-3**(lf)*; *VC*::*hlh-3cDNA*::*GFP* (n = 39).

To address whether *hlh-3* functions cell-autonomously, we assayed expression of a VC differentiation marker *plin-11*::*mCherry* in *hlh-3**(lf)* mutants with a rescuing copy of *hlh-3*. The rescuing extrachromosomal array *icEx274 [VC*::*pes-10*::*hlh-3cDNA*::*GFP*; *pmyo-2*::*mCherry]* was made by introducing a *hlh-3* cDNA into pDM4 (previously shared by Michael Koelle) harboring a VC-specific regulatory region of *lin-11* fused to the basal *pes-10* promoter (Doonan 2014). We find that whereas *hlh-3**(lf)* mutants fail to express the VC differentiation marker *plin-11*::*mCherry* in most VCs, *hlh-3**(lf)* mutants that contain the rescuing extrachromosomal array *icEx27*4 express *plin-11*::*mCherry* in almost all VCs (Figure 7A and 7B). These findings demonstrate that *hlh-3* function is cell-autonomous.

### hlh-3 does not affect the differentiation of other sex-shared neurons in the ventral cord

Given that expression of the *hlh-3* CRISPR-Cas9 edited reporter (*hlh-3*::*gfp*) is detectable in the P cells and its descendants we wished to address whether the absence of *hlh-3* function resulted in defects in the sex-shared neurons. To address this question, we analyzed the expression of cholinergic and GABAergic markers in *hlh-3**(lf)* mutant hermaphrodites. The transcriptional reporter *vsIs48** [punc-17*::*gfp]* gene marks all cholinergic neurons expressing a vesicular acetylcholine transporter (within the VNC this includes VA, VB, AS, DA, DB; Supplemental Figure 1A) (Wormbase: Curatorial remark). The transcriptional reporter *otIs564** [punc-47*::*mChOpti]* marks all GABAergic neurons expressing a vesicular GABA transporter (within the VNC this includes DD and VD neurons; Supplemental Figure 3A) (Gendrel *et al.* 2016). We find that the total number of cholinergic neurons anterior to the vulva is equivalent between WT and *hlh-3**(lf)* individuals (Supplemental Figure 1C). Likewise, the total number of GABAergic neurons is equivalent between WT and *hlh-3**(lf)* hermaphrodites (Supplemental Figure 3B). These analyses demonstrate that the cholinergic and GABAergic sex-shared ventral cord motor neurons acquire their terminal neurotransmitter fate. Thus, *hlh-3* function is not necessary for the acquisition of the terminal fates in sex-shared neurons, rendering its function specific to the terminal differentiation of sex-specific ventral cord VC neurons.

### The male-specific ventral cord motor neurons do not require hlh-3 function

We wondered whether the male-specific ventral cord motor neuron differentiation was also dependent on *hlh-3* function. The CA and CP pairs of male-specific motor neurons arise from the division of the Pn.aap neuroblast, anteriorly (type CA) and posteriorly (type CP) as shown in Supplemental Figure 4A (Sulston *et al.* 1980). We tracked differentiation of the CAs 1-9 and the CPs 1-6 with the differentiation markers for *ida-1* and *tph-1*, respectively (Supplemental Figure 4B). We find that *hlh-3**(lf)* one-day-old adult males, when compared to WT one-day-old adult males show expression of differentiation markers in all CA and CP neurons, nearly at equivalent proportions (Supplemental Figure 4C and 4D). This suggests that *hlh-3* does not have a role in promoting the differentiation of these neurons. Notably, we did not quantify CP0 (descendant of the P2 lineage) although we did observe expression of *pida-1*::*gfp* in both WT and *hlh-3**(lf)* males (data not shown).

## Discussion

### hlh-3 specifies VC fate

Our work identified a new role for the proneural gene *hlh-3* as a regulator of sex-specific motor neuron differentiation in the postembryonic VNC of the hermaphrodite. Both terminal and non-terminal identity features associated with the sex-specific motor neurons, VCs, are reduced or absent in animals that lack *hlh-3* function. We show that VCs do not undergo inappropriate programmed cell death (Figure 6). We confirmed that the VCs are present, in particular the distal VCs, because expression of *otIs619* [*unc-11^promoter 8^*::*NLS*::*mCherry*] is detected in the VCs (Supplemental Figure 5). One-day-old adult hermaphrodites from both genotypes express the pan-neuronal marker at a similar frequency, suggesting that although these cells do not express VC features in *hlh-3**(lf)*, they have a neuronal ground state. While most of our analysis measures transcriptional gene activity of VC identity genes, we also demonstrate that the morphology of the VC subclass is affected. In summary, we implicate *hlh-3* in the specification of the VC motor neuron class; all six VCs require *hlh-3* function.

### Differentiation of the proximal VCs involve hlh-3 dependent and hlh-3 independent mechanisms

**Figure 8:**
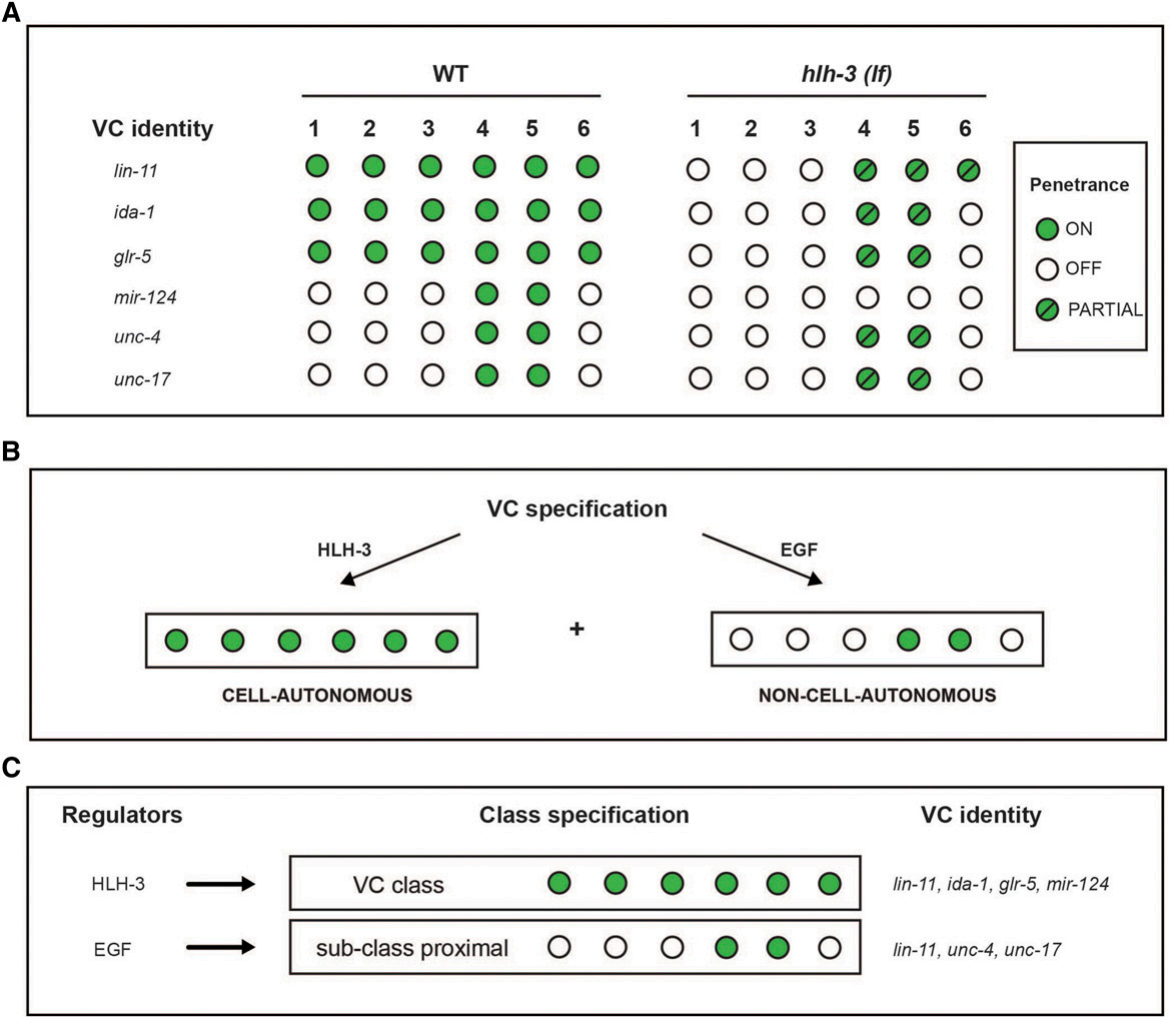
Two pathways promote the acquisition and maintenance of VC-class and VC-subclass features. A: Expression of the VC identity features (*lin-11*, *ida-1*, *glr-5*, *mir-124*) require *hlh-3* function. B: The regulation of the VC identity features occurs in a cell-autonomous way prior and independently of EGF signaling during mid-L4 development. The alternative pathway, dependent on EGF, regulates the expression of *unc-4* (Zheng *et al.* 2013). We propose that the function of EGF signaling adds a secondary input to regulate *lin-11* levels in the proximal VCs, and affect *unc-4* and *unc-17*, as well as other VC identity features.

Our analysis also revealed that in the absence of *hlh-3* function, the differentiation of proximal VCs is less affected than that of distal VCs. We have gained some insight into these differences with the analysis of markers that are expressed in early L4 *vs.* later L4 substages (Figure 3). Expression of VC class and VC subclass-specific identity features *ida-1*, *glr-5*, and *mir-124*, is seen in VCs in early L4 substages in a WT context, yet, is completely absent from these early substages through adulthood in animals that lack *hlh-3* function (Figure 3; Figure 5; Figure 8A; Supplemental Figure 2). This indicates *hlh-3* function is required in all six VCs before L4 development through adulthood. However, our findings also support that a *hlh-3* independent mechanism regulates expression of features in the proximal subclass (see below). Lastly, our findings support that *hlh-3* function is necessary for expression of VC features but it does not address whether the role is a direct one.

As mentioned before, in the course of our studies we also learned that in the mutant context, and during later stages of L4 development, expression of VC identity features appeared in just a few VCs, the proximal ones. This suggests that there may be a parallel pathway, which can promote VC differentiation. Since the proximal VCs are less affected in their expression of the terminal identity genes that arise after mid-L4 development (*unc-4* and *unc-17*), we propose that this alternative pathway acts by mid L4 but not sooner. We infer that the *hlh-3* independent parallel pathway is mediated by EGF, a cue secreted by vulF cells as early as mid L4, already shown to be required for expression of *unc-4* in proximal VCs (Figure 8B; Zheng *et al.* 2013). The presence of this parallel pathway could ensure that at least proximal VCs retain some function, as they are primary contributors to egg laying by providing feedback to HSNs and vulva muscles (Schafer 2006).

In summary, we have found that the acquisition of VC class features (shown herein) is impaired in *hlh-3**(lf)* individuals. Of the features we have analyzed, only one subclass differentiation feature, expression of *mir-124*, is fully dependent on *hlh-3* function (Figure 5C; Figure 8A). Since *mir-124* expression is restricted to the VC proximal subclass, it may have a role in promoting VC subclass diversity. However, since the expression of *mir-124*, is seen prior to the EGF cue, and is completely absent in *hlh-3**(lf)*, we believe that it is regulated by *hlh-3* and not by the EGF-dependent pathway. Further work has to address whether *mir-124* functions as an intrinsic, cell-autonomous mechanism to promote VC class diversity.

With this work, we propose that: (1) *hlh-3* functions cell-autonomously to specify VC class fate in development (from L1 to adulthood) and (2) during L4 development an EGF-dependent cue promotes proximal VC subclass fate diversity for function in egg laying. Our proposal is consistent with the observation that expression of *lin-11*, *glr-5*, *ida-1*, and *unc-4*, in the proximal VCs, is not significantly altered in the absence of *hlh-3* function. To reiterate, the proposed *hlh-3* dependent pathway specifies VC class fate and an *hlh-3* independent pathway promotes VC subclass diversity.

### The LIM homeodomain transcription factor LIN-11 in VCs is downstream of and positively regulated by hlh-3

As shown by others, the gene encoding LIN-11 is expressed from L2 through adulthood (Hobert *et al.* 1998). We have observed this as well with the translational reporter *wgIs62** (lin-11*::*TY1*::*EGFP*::*3xFLAG + **unc-119**(+))* (data not shown). Since our analysis indicates that *hlh-3* is expressed before *lin-11*, we characterized the expression of a different *lin-11* transcriptional reporter *(plin-11*::*mCherry)* in the absence of *hlh-3* function. We showed that *hlh-3**(lf)* mutants exhibit reduced *lin-11* transcriptional activity in VCs (Figure 7). It is likely that *hlh-3* directly targets *lin-11*, but further work will determine whether this effect is direct or indirect. Interestingly, the ortholog ASCL1 has been shown to directly target the *lin-11* ortholog, Lhx1, in a ChIP-seq analysis of the ventral telencephalon (Raposo *et al.* 2015; Kim *et al.* 2018).

Our analysis of *lin-11* expression in *hlh-3**(lf)* also revealed that the proximal VCs are less affected than the distal VCs by the absence of *hlh-3* function (Figure 7; Figure 8A). The proximal VCs express *plin-11*::*mCherry* at higher proportions than the distal VCs. Thus, the presence of *lin-11* transcriptional activity may be dependent on a secondary pathway other than one that is mediated by *hlh-3*. Given that others have shown *lin-11* acts downstream of EGF, *lin-11* may be targeted by both a *hlh-3* dependent pathway and this secondary EGF-dependent pathway (Figure 8B; Zheng *et al.* 2013).

We propose that the reason *lin-11* transcriptional activity is observed in the proximal VCs of *hlh-3**(lf)* individuals is that EGF-dependent signaling is acting in parallel to *hlh-3*. It is known that the proximal VCs acquire this subclass-specific identity feature (*unc-4*) in a time-dependent manner, occurring after EGF signaling, after mid-L4 development (Zheng *et al.* 2013). Our analysis suggests the EGF signaling pathway promotes *lin-11* transcription too. This would explain why, in the absence of *hlh-3*, there is still expression of *lin-11* (Figure 7). Lastly, our findings that *hlh-3**(lf)* mutants also exhibit reduced *unc-4* transcriptional activity in the proximal VCs is a logical consequence of lower *lin-11* expression in the proximal VCs (Figure 4E; Figure 8A). Our model shows that two pathways affect the expression of *lin-11* and other VC identity genes (Figure 8).

### hlh-3 may be a terminal selector of VC fate

Terminal selectors are factors that initiate and maintain the expression of effector genes, required in the final determination of neuronal subtype specification (Allan and Thor 2015). *hlh-3* meets several criteria to be classified as a gene encoding a terminal selector in the VCs. First, it is expressed from the birth to the maturation of all VC features. Second, in its absence, all known VC class terminal identity features fail to be acquired. Lastly, it functions cell-autonomously. Since more than one terminal selector can function to regulate downstream effector genes, it is possible that another terminal selector may function with *hlh-3*. To confirm if *hlh-3* is a terminal selector, additional work will need to test for the direct regulation of VC identity genes by *hlh-3*.
